# Anauralia: The Silent Mind and Its Association With Aphantasia

**DOI:** 10.3389/fpsyg.2021.744213

**Published:** 2021-10-14

**Authors:** Rish P. Hinwar, Anthony J. Lambert

**Affiliations:** School of Psychology and Centre for Brain Research, University of Auckland, Auckland, New Zealand

**Keywords:** anauralia, aphantasia, auditory imagery, visual imagery, sensory imagery, imagery, consciousness, cognition

## Abstract

Auditory and visual imagery were studied in a sample of 128 participants, including 34 self-reported aphantasics. Auditory imagery (Bucknell Auditory Imagery Scale-Vividness, BAIS-V) and visual imagery (Vividness of Visual Imagery Questionnaire-Modified, VVIQ-M) were strongly associated, Spearman's *rho* = 0.83: Most self-reported aphantasics also reported weak or entirely absent auditory imagery; and participants lacking auditory imagery tended to be aphantasic. Similarly, vivid visual imagery tended to co-occur with vivid auditory imagery. Nevertheless, the aphantasic group included one individual with typical auditory imagery; and the group lacking auditory imagery (*N* = 29) included one individual with typical visual imagery. Hence, weak visual and auditory imagery can dissociate, albeit with low apparent incidence. Auditory representations and auditory imagery are thought to play a key role in a wide range of psychological domains, including working memory and memory rehearsal, prospective cognition, thinking, reading, planning, problem-solving, self-regulation, and music. Therefore, self-reports describing an absence of auditory imagery raise a host of important questions concerning the role of phenomenal auditory imagery in these domains. Because there is currently no English word denoting an absence of auditory imagery, we propose a new term, *anauralia*, for referring to this, and offer suggestions for further research.

## Introduction

Interest in the once controversial topic of mental imagery (Pylyshyn, 2002), and its relation with other aspects of psychological functioning has revived in recent years, following a series of papers describing individuals who apparently have no experience of imagery, or more accurately no experience of visual sensory imagery (Zeman et al., 2010, 2015, 2020; Dawes et al., 2020). The term “aphantasia” was introduced by Zeman et al. (2015) to describe this lack of visual imagery. However, in addition to reporting a lack of visual imagery, some but not all aphantasic individuals also report weak or absent imagery in other sensory modalities (Dawes et al., 2020; Zeman et al., 2020). While humans are undoubtedly a highly visual species, other sensory modalities are obviously also important. In particular, auditory representations and auditory imagery, including the notion of an “inner voice” are thought to be critically important for psychological functioning across a wide range of domains (Reisberg, 1992; Hubbard, 2010; Alderson-Day and Fernyhough, 2015; Fernyhough, 2016), including: working memory and memory rehearsal (Baddeley and Logie, 1992; Baddeley and Andrade, 2000), prospective memory (Stone et al., 2001), language (Vygotsky, 1962), reading (Kurby et al., 2009; Brunye et al., 2010), planning and problem-solving (Morin et al., 2018), self-regulation (Tullett and Inzlicht, 2010), thinking (Vygotsky, 1962; Clowes, 2007; Chella and Pipitone, 2020) and music (Aleman et al., 2000). Moreover, auditory imagery in the form of inner speech is believed to play a key role in cognitive development (Vygotsky, 1962; Alderson-Day and Fernyhough, 2015). Just as the literature on mental imagery generally has been dominated by work on visual imagery (Pearson, 2019), descriptions and investigations of aphantasia have tended to focus on visual representations and visual imagery. However, both personal accounts (Faw, 2009; Kendle, 2017; Watkins, 2018) and survey studies (Dawes et al., 2020; Zeman et al., 2020) have shown that at least some aphantasics report an inner mental life that is not only “blind” (Keogh and Pearson, 2018), but also completely silent. That is, these individuals report a complete absence of auditory as well as visual imagery: “*I just don't have an inner voice that speaks to me or which I can listen to talking”—*Kendle (2017, p. 14); “*I silently think and silently read (with no auditory ‘voice’)”*—Faw (2009, p. 46); “*I don't have the experience people describe of hearing a tune or a voice in their heads”*—Watkins (2018, p. 44); “*I now refer to my experience as “Like Helen Keller in my head. I'm blind, deaf, dumb and mute!”—*Kendle (2017, p. 38). Because there is currently no English word that denotes an absence of auditory imagery, we propose a new term, *anauralia*, to refer to this. Since auditory representations are believed to be important for key aspects of cognitive functioning as we have noted, further investigation of auditory imagery, its absence in anauralia, and the relationship between anauralia and aphantasia appears overdue. Accordingly, in this paper, we report a preliminary investigation of auditory imagery and anauralia and their associations with visual imagery and aphantasia.

Two recent survey studies have furnished data on the extent to which individuals described as aphantasic, experience imagery in other sensory modalities. Dawes et al. (2020) used the short form of Betts' Questionnaire upon Mental Imagery (Sheehan, 1967) to assess imagery across seven sensory domains (visual, auditory, tactile, kinesthetic, taste, olfactory, emotion) in 267 self-reported aphantasics. Most of these individuals (73.8%) reporting experiencing some degree of imagery in non-visual sensory modalities, including audition. However, vividness ratings for non-visual imagery were substantially lower for aphantasic compared to control participants. Within the aphantasic group, while ratings of visual imagery were essentially at “floor,” corresponding to complete absence of imagery, average ratings of non-visual (including auditory) imagery corresponded to weak, but not entirely absent imagery. In another recent survey study, Zeman and colleagues found that 35.8% of aphantasic respondents reported normal or vivid imagery in at least one other (i.e., non-visual) sensory modality, while 54.2% reported weak or absent imagery in all sensory modalities (Zeman et al., 2020).

In addition to evidence from survey research, some personal accounts of aphantasic individuals describe a mental life that includes imagery in other sensory modalities, including auditory imagery and an “inner voice” (Kendle, 2017). As noted above, others report a complete absence of auditory imagery (i.e., anauralia; Faw, 2009; Kendle, 2017; Watkins, 2018), or weak auditory imagery (Ross, 2016).

Halpern (2015) studied visual and auditory imagery in a sample of 76 college students, and found a moderate-to-strong association between self-rated vividness of visual and auditory imagery (Pearson's *r* = 0.62). However, it is worth noting that in this study, participants' scores on a modified version of the Vividness of Visual Imagery Questionnaire (VVIQ-M), (Marks, 1973; McKelvie, 1995; Halpern, 2015) ranged from 3.0 to 7.0 on a 7-point Likert scale. Using criteria adopted by Dawes et al. (2020) and Zeman et al. (2020), none of the individuals included in the study of Halpern (2015) would be categorised as aphantasic. Hence, while the data of Halpern (2015) shed light on the relationship between visual and auditory imagery among those who experience average or better than average visual imagery, they provide no information concerning the experience of auditory imagery in aphantasia.

Thus, in the literature to date, detailed information concerning relationships of anauralia and auditory imagery with aphantasia and visual imagery is lacking. Accordingly, the current study investigated auditory imagery in a sample that included aphantasic participants, who are likely to experience reduced imagery in both the visual and auditory domains. The study sample also included individuals reporting average, and more vivid than average visual imagery. We aimed to evaluate possible associations and dissociations between visual and auditory imagery, and their absence.

While the presence of statistically reliable associations between variables is a central focus in many areas of psychology, in cognitive neuroscience documenting the presence of dissociations, often through single-case studies (e.g., see Ganel and Goodale, 2017), can be an equally important source of theoretical insight. Therefore, in addition to examining group-level associations between visual and auditory imagery, dissociations were also noted. That is, we evaluated the proportion of aphantasic individuals who also reported anauralia; and the proportion who reported average or more vivid than average auditory imagery. The converse proportions were also evaluated: i.e., the number of anauralic individuals who also reported aphantasia, and who reported average or more vivid than average visual imagery.

## Method

### Participants

Potential participants were recruited through several avenues. Firstly, information concerning the project, together with invitations to participate were circulated among three Facebook groups entitled: “Aphantasia!,” “Aphantasia Support Group,” and “Aphantasia (Non-Imager/Mental Blindness) Awareness Group.” Each Facebook group advertised itself as a social group for individuals with self-proclaimed low visual mental imagery. Secondly, information about the project, together with an invitation to participate was circulated digitally to undergraduate psychology classes, and via physical advertising on notice-boards at the University of Auckland. Thirdly, project information and an invitation to participate was circulated to social media contacts of one of the researchers (RH). 197 individuals responded to these invitations by commencing the survey; 128 participants (86 female, 40 male, 2 gender diverse) completed all survey items successfully. All participants were over 18 years, with the majority (*N* = 84) being 18–29 years. Ages of the remaining participants were 30–49 years (*N* = 30), 50–69 years (*N* = 13) and over 70 years (*N* = 1).

### Procedure

Participants responded to an invitation to participate by accessing an online survey presented *via* Qualtrics^tm^ software. After completing an initial item confirming participant age as 18 years or over, and indicating informed consent, participants provided demographic information concerning gender, age and whether they resided in Auckland, New Zealand. Following this, participants completed three previously described instruments, assessing auditory and visual sensory imagery.

### Instruments

Participants completed the 14-item Bucknell Auditory Imagery Scale—Vividness (BAIS-V—see Halpern, 2015), followed by the 14-item Bucknell Auditory Imagery Scale—Control (BAIS-C, see Halpern, 2015), followed by the 16-item Vividness of Visual Imagery Questionnaire (VVIQ-M—see Marks, 1973, McKelvie, 1995, Halpern, 2015). 7-point Likert scales were employed for all three imagery questionnaires, with the same anchors and similar question format to that adopted by Halpern (2015). Minor modifications were introduced to the wording of several BAIS-V and BAIS-C items, due to cultural differences between New Zealand and North America (e.g., imagined scenarios involving baseball games were replaced with scenarios involving rugby games). Full versions of all three imagery questionnaires can be found in the Supplementary Material and at: https://data.mendeley.com/datasets/p32vxydy3r/1

## Results

### Visual and Auditory Imagery Vividness Scores

Across the entire sample (*N* = 128), descriptive statistics for visual and auditory imagery vividness were: Mean VVIQ-M = 4.23, 95% CI (with bootstrapping) = 3.90–4.58, SD = 2.09; Mean BAIS-V = 4.05, 95% CI (with bootstrapping) = 3.73–4.38, SD = 1.92. Raw data can be found in the Supplementary Material.

Participants with mean VVIQ-M scores of 2.0 or less were categorised as aphantasic. This is similar to the criteria employed by Dawes et al. (2020) and Zeman et al. (2020), but slightly more conservative due to our adoption, following, Halpern (2015), of a 7-point, rather than a 5-point Likert scale for assessing both visual and auditory imagery. Similarly, participants with mean BAIS-V scores of 2.0 or less were categorised as experiencing anauralia. At the upper end of the score distributions, participants with mean VVIQ-M / BAIS-V scores of 6.0 or greater were categorised as experiencing hyperphantasia or hyperauralia, respectively. In addition, VVIQ-M and BAIS-V scores were categorised as reflecting weak (2–4) or average (4–6) imagery. The average imagery categories were centred approximately on the mean BAIS-V values (mean = 5.1, SD = 0.9), and mean VVIQ-M values (mean = 5.4, SD = 1.0) observed by Halpern (2015). Table 1 provides a complete cross-tabulation of the number of participants in each visual and auditory imagery sub-group.

**Table 1:** Visual and auditory imagery vividness.

	**Anauralia** ***(BAIS-V ≤ 2)***	**Weak Aud. Imagery** ***(BAIS-V 2-4)***	**Average Aud. Imagery** ***(BAIS-V 4-6)***	**Hyperauralia** ***(BAIS-V ≥ 6)***	**Totals**
Aphantasia (*VVIQ-M ≤ 2)*	**28**	5	1	0	**34**
Weak Visual Imagery *(VVIQ-M 2-4)*	0	**5**	6	0	**11**
Average Visual Imagery *(VVIQ-M 4-6)*	1	10	**42**	3	**56**
Hyperphantasia *(VVIQ-M ≥ 6)*	0	1	11	**15**	**27**
**Totals**	**29**	**21**	**60**	**18**	**128**

*Bolding these values merely highlights for the reader associations between the four auditory imagery categories and the four visual imagery categories*.

### Aphantasia and Anauralia Are Associated

Using the above criteria, 34 of our total sample of 128 participants were categorised as aphantasic; and 29 were categorised as anauralic. These two groups overlapped to a large extent. As a group, the aphantasic individuals experienced very weak auditory imagery (Mean BAIS-V = 1.42, SD = 0.95), with 82% also being categorised as anauralic. Similarly, the anauralic group reported very weak visual imagery (Mean VVIQ-M = 1.19, SD =0.88), with 97% also being categorised as aphantasic. As one would expect, given these observations, the association between aphantasia and anauralia was highly reliable, χ^2^ = 93.42, df = 1, *p* < 0.001.

### Hyperphantasia and Hyperauralia Are Associated

27 participants were categorised as hyperphantasic; and 18 were categorised as hyperauralic (mean VVIQ-M / BAIS-V score ≥ 6). Once again, there was substantial overlap between these classifications. As a group, the hyperphantasic individuals also experienced strong auditory imagery (Mean BAIS-V = 6.01, SD = 0.75), with 56% also being categorised as hyperauralic. Similarly, the hyperauralic group reported strong visual imagery (Mean VVIQ-M = 6.40, SD = 0.55), with 83% also being categorised as hyperphantasic. As one would expect, given these observations, the association between hyperphantasia and hyperauralia was highly reliable, χ^2^ = 48.37, df = 1, *p* < 0.001.

### Dissociations Between Visual and Auditory Imagery

1/34 aphantasic individuals reported average auditory imagery (Mean BAIS-V = 5.0); and 1/29 anauralic individuals reported average visual imagery (Mean VVIQ-M = 5.75). Hence, it appears that auditory imagery can dissociate to some extent from visual imagery in aphantasia; and visual imagery can dissociate to some extent from auditory imagery in anauralia, albeit with low incidence in each case. On the other hand, strong dissociations were not observed. In the current sample, no aphantasic participants were hyperauralic; and no anauralic participants were hyperphantasic.

**Figure 1:**
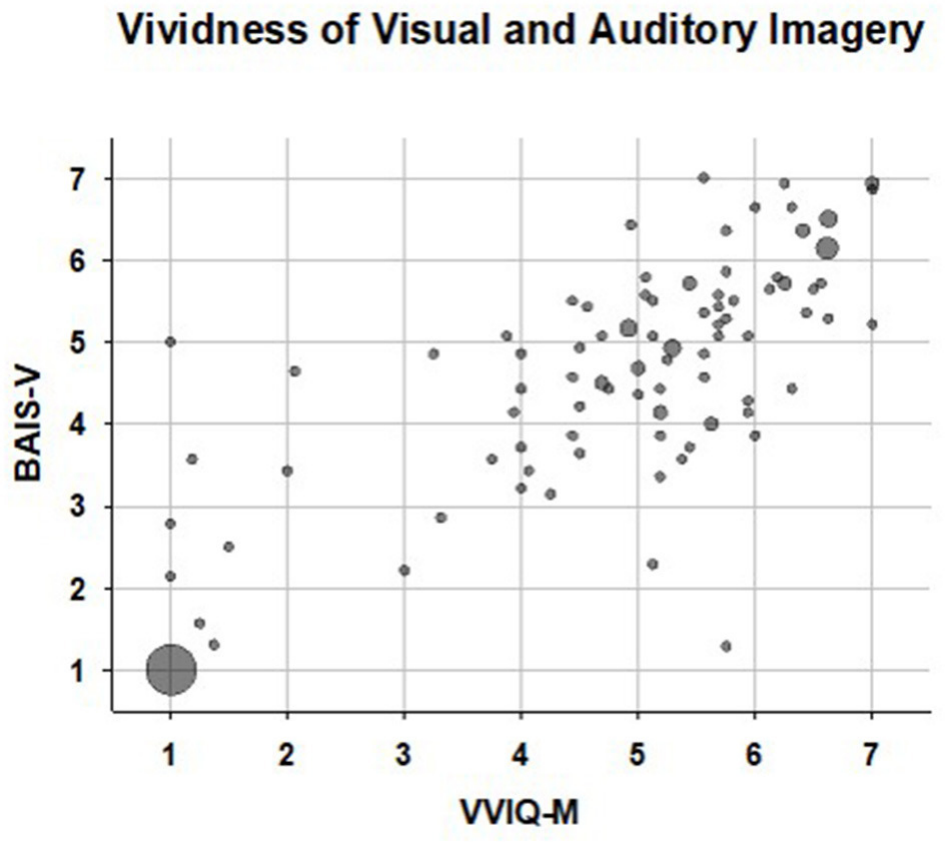
Bubble plot of vividness of visual (VVIQ-M) and auditory (BAIS-V) imagery scores. Larger bubbles represent participants with identical, or near identical (differing by 0.2 or less) VVIQ-M and BAIS-V scores. The large bubble at the lower left represents the twenty six participants reporting a total, or near total (average score < 1.2) absence of both visual and auditory imagery. The data point in the upper left represents the single participant who reported typical auditory imagery in the context of aphantasia; the data point in the lower right represents the single participant who reported typical visual imagery in the context of anauralia.

A bubble plot illustrating the relationship between rated vividness of visual (VVIQ-M) and auditory (BAIS-V) imagery is shown in Figure 1. Participants (*N* = 26) with scores representing extremely weak or absent visual imagery (mean VVIQ-M < 1.2) and extremely weak (or absent) auditory imagery (mean BAIS-V < 1.2) are represented by the large bubble in the lower left of this figure. Notably, there were no fewer than 24 participants with mean VVIQ-M = 1.00 and mean BAIS-V scores = 1.00. That is, these participants indicated the extreme value “1—no image present at all” for all 16 VVIQ-M items, and all 14 BAIS-V items. Data points representing the one individual with average auditory imagery in the context of aphantasia, and the individual with average visual imagery in the context of anauralia, are apparent in the upper-left and lower right portions of Figure 1 respectively.

Because our recruitment strategy targeted aphantasia interest groups, the distribution of VVIQ scores was non-normal, with a clear mode at the lower extreme. The distribution of BAIS-V scores was also non-normal, with a similar mode at the lower extreme of the distribution. Accordingly, a non-parametric statistic, Spearman's *rho*, was used to estimate the association between visual and auditory imagery. This association was strong: Spearman's *rho* = 0.832, 95% CI (with bootstrapping) = 0.743–0.897, *p* < 0.001.^1^

## Discussion

As Figure 1 and Table 1 illustrate, visual and auditory imagery were strongly associated (Spearman's *rho* =0.83). A large majority of self-reported aphantasics were also anauralic, and vice-versa; and most hyperphantasics were also hyperauralic, and vice-versa. A salient feature of the data is the number of participants who reported a complete or near-complete absence of auditory imagery, meriting inclusion in the anauralia category. When recruiting participants for this study we sought to include those with weak imagery, but of necessity this involved targeting individuals with weak visual imagery, via aphantasia online interest groups. Because anauralia is a new term, online interest groups concerned specifically with weak or absent auditory imagery do not exist. Nevertheless, a substantial number of our participants reported a complete or near-complete absence of auditory imagery: 29 were categorised as anauralic, of whom 25 indicated the extreme minimum option (“1- no image present at all”) for all 14 BAIS-V items. In the literature to date, investigations of aphantasia have, perhaps unsurprisingly, emphasised associations of visual imagery and its absence with other aspects of psychological functioning. For example, aphantasia has been linked with poor autobiographical memory (AM) and face recognition problems; and aphantasics appear more likely to follow scientific and technical occupations (Watkins, 2018; Dawes et al., 2020; Zeman et al., 2020). However, the current data highlight the strength of the association between lack of visual imagery and lack of auditory imagery; and as noted earlier, auditory imagery and auditory representations are thought to play important roles in a wide range of cognitive processes. Therefore, it is unclear whether associations between aphantasia and other psychological processes, such as autobiographical memory reflect causal influences of visual imagery and its absence; the current findings underline the plausibility of alternative interpretations. For example, the association between aphantasia and poor AM could be driven by a causal link between deficient visual imagery and AM, and/or between deficient auditory imagery and AM, and/or between deficient sensory imagery generally and AM, and/or between AM and a further (unknown) factor associated with poor sensory imagery. Interpreting associations between aphantasia and other aspects of psychological function (Dawes et al., 2020; Zeman et al., 2020) are subject to the same caveat. However, the substantial literature cited earlier, linking auditory imagery and auditory representations with a wide range of cognitive functions underlines the plausibility of the hypothesis that lack of auditory imagery (i.e., anauralia) may play a significant role in at least some of the associations observed in the aphantasia literature, especially in the domains of memory and prospective cognition (Watkins, 2018; Dawes et al., 2020; Zeman et al., 2020). Testing this hypothesis will require large-scale studies that disentangle specific associations of aphantasia and anauralia with cognitive functioning and other psychological characteristics.

An association between auditory and visual imagery, including both their absence in anauralia and aphantasia and their abundance in hyperauralia and hyperphantasia, is broadly consistent with neuroimaging work. When participants perform multisensory imaging tasks, complex network activations involving multiple brain regions have been observed. Some of these activations appear to reflect modality-specific activation of sensorimotor representations (Yoo et al., 2001; McNorgan, 2012), while other activations appear to reflect supramodal mechanisms involved in generating sensory imagery regardless of modality (McNorgan, 2012; Kleider-Offutt et al., 2019). In an important study, Lima et al. (2015) studied individual differences in sensory imagery, and investigated associations between auditory and visual imagery, and their relationships with brain structure and function. In common with the current study, auditory and visual imagery vividness were associated. Interestingly, grey matter volume in the supplementary motor area (SMA) was associated with individual differences in both auditory and visual imagery vividness (Lima et al., 2015).

Variations in survey methodology make direct comparisons between the current results and earlier survey-based findings difficult. Zeman et al. (2020) asked aphantasic and hyperphantasic participants to indicate whether lack, or abundance of visual imagery respectively, affected imagery in other sensory modalities, without referring to any specific modality or modalities. Therefore, this study cannot be compared directly with the current investigation, which focused specifically on links between visual and auditory imagery. Nevertheless, Zeman et al. (2020) reported that 35.8% of aphantasic participants experienced normal imagery in at least one other modality. This contrasts with the current data, indicating that only 17.6% of aphantasics experienced auditory imagery in the weak to average range, and just 2.9% (i.e., 1/34) experienced average auditory imagery. No aphantasics reported experiencing strong auditory imagery. Hence, while the data gathered by Zeman et al. (2020) suggest a moderate association between aphantasia and imagery in all other modalities, the current data highlight the strength of the association between visual and auditory imagery.

In common with the current investigation, the study of, Dawes et al. (2020) did include items specifically about auditory imagery, and like the current study, participants were asked to rate the vividness of auditory images on a 7-point Likert scale. However, the inventory used by Dawes et al. (2020) comprised the five auditory items of the Short Form of Betts' Questionnaire upon Mental Imagery (Sheehan, 1967), while the current study employed the 14-item BAIS-V, so again, direct comparisons are difficult. However, in both the current study and Dawes et al. (2020) ratings of auditory image vividness were substantially lower for aphantasic compared to non-aphantasic individuals, and corresponded to weak auditory imagery. In the current study, mean auditory imagery of the aphantasic group was less than the criterial value (2) for anauralia [mean = 1.42, 95% CI (with bootstrapping) = 1.15–1.77, SD = 0.95].

Although our group-level analyses have highlighted the strength of the association between visual and auditory imagery, dissociations were also evident, albeit with low incidence. Within the aphantasic group, a single individual experienced average auditory imagery; and within the anauralic group one individual reported average visual imagery. From a cognitive neuroscience perspective, rather than discarding these cases as “statistical outliers,” the existence of such dissociations can be valuable and theoretically informative. While the presence of a strong association suggests that common mechanisms are involved when generating visual and auditory images (Lima et al., 2015; Kleider-Offutt et al., 2019), the existence of dissociations indicates that use of common mechanisms and pathways is not mandatory, or that the degree of overlap is less than complete. On the other hand, strong dissociations were not observed—the number of aphantasics who were hyperauralic, and the number of anauralics who were hyperphantasic was zero in both cases. Clearly, further and larger-scale studies will be required, to gain more comprehensive information about the relative incidence of moderate and strong dissociations between visual and auditory imagery.

Exclusive reliance on self-reports of internal phenomenal experience is a limitation of this, and other studies of sensory imagery (de Vito and Bartolomeo, 2016; Zeman et al., 2016). The large number of participants who selected the left-hand anchor (“1—no image present at all”) for all 14 items of the BAIS-V and all 16 items of the VVIQ-M raises the possibility that participants may have been responding automatically, selecting the same option for each and every question, without attending to its specific content. While this possibility cannot be ruled out entirely, it is worth noting that the BAIS-V items were the first imagery items to be presented, after the initial consent and demographic items. The primacy of these items makes it unlikely that participants categorised as anauralic were merely responding in an automatic fashion, due to repeated use of the same response option. Concerns over the central role of self-report measures in aphantasia research have also been raised by de Vito and Bartolomeo (2016). However, important work by Keogh and Pearson (2018) has shown that self-assessment of aphantasia is associated with an objective behavioural measure—imagery-based priming in a binocular rivalry paradigm. Development of an analogous auditory priming measure, to supplement self-reports of auditory imagery and its absence, would be a valuable innovation for future investigations of anauralia.

## Conclusion

As others have noted, the literature on sensory imagery has been dominated by work on visual sensory imagery, with the important role played by auditory imagery arguably receiving less attention than it deserves (Reisberg, 1992; Hubbard, 2010). Similarly, both the research literature and personal accounts of aphantasia have tended to foreground visual imagery and its absence. However, it appears that aphantasia tends to co-occur with anauralia, and an extensive literature attests to the important roles played by auditory imagery and auditory representations in many aspects of psychological functioning, including memory, prospective cognition, thinking, reading, planning, problem-solving, self-regulation, and music The phenomenon of anauralia, documented here, raises a host of important questions concerning inter-relationships between auditory representations, phenomenal auditory imagery and cognitive functioning. Therefore, it may be time refocus, or perhaps broaden the focus of research efforts in this field, to investigate anauralia and its relationships with a range of cognitive functions, especially in the realms of working and autobiographical memory and prospective cognition. Disentangling specific associations of anauralia and aphantasia with cognitive functioning will require large-scale investigations.

## Data Availability Statement

The datasets presented in this study can be found in online repositories. The names of the repository/repositories and accession number(s) can be found in the article/Supplementary Material.

## Ethics Statement

The studies involving human participants were reviewed and approved by University of Auckland Human Participants Ethics Committee. The patients/participants provided their written informed consent to participate in this study.

## Author Contributions

The initial conception and design of the study was provided by AL. RH contributed to the design and selected the questionnaire measures. The data were collected by RH. RH and AL analysed the data. Both authors contributed to writing the manuscript, with AL taking the primary role. AL coined the term *anauralia*. All authors contributed to the article and approved the submitted version.

## Conflict of Interest

The authors declare that the research was conducted in the absence of any commercial or financial relationships that could be construed as a potential conflict of interest.

## Publisher's Note

All claims expressed in this article are solely those of the authors and do not necessarily represent those of their affiliated organizations, or those of the publisher, the editors and the reviewers. Any product that may be evaluated in this article, or claim that may be made by its manufacturer, is not guaranteed or endorsed by the publisher.

## Footnotes

^1^Participants also completed the 14-item Bucknell Auditory Imagery Scale—Control (BAIS-C—see Method). The rationale for collecting these data arose from the aims of a further investigation, in which some of our Auckland-based participants were invited to visit our laboratory to take part in a study which involved measuring changes in eye pupil size under different experimental conditions. Although BAIS-C data were tangential to the aim of the current study, the association between VVIQ-M and BAIS-C scores was nevertheless evaluated, for the sake of completeness. This association was reliable: Spearman's *rho* = 0.776, 95% CI (with bootstrapping) = 0.652–0.868, *p* < 0.001. As others have reported (Halpern, 2015), BAIS-V and BAIS-C scores were strongly associated: Spearman's *rho* = 0.810, 95% CI (with bootstrapping) = 0.692–0.892, *p* < 0.001.

## Supplementary Material

Supplementary Table 1 Imagery survey results.

Supplementary Data Sheet 1 Copy of imagery survey.
