# Supplementary material for: Anauralia: The Silent Mind and Its Association With Aphantasia
Source: Front Psychol. 2021 Oct 14;12:744213. doi: 10.3389/fpsyg.2021.744213 (PMC8551557; doi:10.3389/fpsyg.2021.744213)
Supplement: Supplementary Data Sheet 1 — Copy of imagery survey. [file Data_Sheet_1.PDF]

# Mental Imagery Questionnaire

Start of Block: Captcha Item

Please tick the CAPTCHA below.

End of Block: Captcha Item

Start of Block: Informed Consent

Welcome to the Visual and Auditory Imagery Study

We are interested in understanding mental imagery. When you think of Jacinda Ardern or Winston Peters, how clearly can you imagine their appearance and hairstyles? How clearly can you imagine the sound of their voices? The aim of this study is investigate the relationship between imagining a visual scene, and imagining sounds. Do individuals who report experiencing vivid visual imagery in their 'mind's eye' also experience vivid auditory imagery, in their 'mind's ear'? Or are these two kinds of mental imagery unrelated? You will be presented with a series of questions concerned with imagining visual scenes and imagining sounds. You will also be asked to provide information about your age and gender. Please be assured that your responses and the information you provide will be kept completely confidential.

The study should take you around 15 minutes to complete. Your participation in this research is voluntary. You have the right to withdraw at any point during the study, for any reason, and without any prejudice. If you would like to contact the Principal Investigator in the study to discuss this research, please e-mail: Professor Anthony Lambert, School of Psychology, University of Auckland (a.lambert@auckland.ac.nz).

This research project has been approved by the University of Auckland Human Participants Ethics Committee.

By clicking the button below, you acknowledge that your participation in the study is voluntary, you are 18 years of age, and that you are aware that you may choose to terminate your participation in the study at any time and for any reason.

Please note that this survey will be best displayed on a laptop or desktop computer. Some features may be less compatible for use on a mobile device.

- ○ I consent, begin the study (1)
- ○ I do not consent, I do not wish to participate (2)

*Skip To: End of Survey If Welcome to the Visual and Auditory Imagery Study We are interested in understanding mental ima... = I do not consent, I do not wish to participate*

End of Block: Informed Consent

The first part of the questionnaire will ask you about some demographic information. Please select your answer by ticking the box to the right of the option that applies to you.

---

Q1 What is your gender?

- ○ Male (1)
- ○ Female (2)
- ○ Other (3)
- ○ Prefer not to say (4)

---

Q2 What is your age?

- ○ 18 - 29 (1)
- ○ 30 - 49 (2)
- ○ 50 - 69 (3)
- ○ 70+ (4)

---

Q3 Do you currently live in Auckland, New Zealand?

- ○ Yes (4)
- ○ No (5)

**End of Block:**

---

Start of Block: The Bucknell Auditory Imagery Scale - Vividness (BAIS-V)

Q1 The following scale is designed to measure auditory imagery, or the way in which you “think about sounds in your head.” For the following items you are asked to do the following: Read the item and consider whether you think of an image of the described sound in your head. Then rate the vividness of your image using the following “Vividness Rating Scale.” If no image is generated, give a rating of 1.

Please feel free to use all of the levels in the scale when selecting your ratings.

For the first item, consider the beginning of the song "Happy Birthday." Try to imagine the sound of a trumpet beginning the piece.

- ◯ 1 - No Image Present at All (1)
- ◯ 2 (2)
- ◯ 3 (3)
- ◯ 4 (4)
- ◯ 5 (5)
- ◯ 6 (6)
- ◯ 7 - As Vivid as the Actual Sound (7)

---

Q2 For the next item, consider ordering something over the phone. Try to imagine the voice of an elderly clerk assisting you.

- 1 - No Image Present at All (1)
- 2 (2)
- 3 (3)
- 4 (4)
- 5 (5)
- 6 (6)
- 7 - As Vivid as the Actual Sound (7)

---

Q3 For the next item, consider being on the beach. Try to imagine the sound of the waves crashing against nearby rocks.

- 1 - No Image Present at All (1)
- 2 (2)
- 3 (3)
- 4 (4)
- 5 (5)
- 6 (6)
- 7 - As Vivid as the Actual Sound (7)

---

Q4 For the next item, consider going to a dentist appointment. Try to imagine the loud sound of the dentist's drill.

- ◯ 1 - No Image Present at All (1)
- ◯ 2 (2)
- ◯ 3 (3)
- ◯ 4 (4)
- ◯ 5 (5)
- ◯ 6 (6)
- ◯ 7 - As Vivid as the Actual Sound (7)

---

Q5 For the next item, consider being present at a nightclub. Try to imagine the sound of music playing.

- ◯ 1 - No Image Present at All (1)
- ◯ 2 (2)
- ◯ 3 (3)
- ◯ 4 (4)
- ◯ 5 (5)
- ◯ 6 (6)
- ◯ 7 - As Vivid as the Actual Sound (7)

---

Q6 For the next item, consider being at a live rugby game. Try to imagine the cheers of the crowd as a player scores.

- ○ 1 - No Image Present at All (1)
- ○ 2 (2)
- ○ 3 (3)
- ○ 4 (4)
- ○ 5 (5)
- ○ 6 (6)
- ○ 7 - As Vivid as the Actual Sound (7)

---

Q7 For the next item, consider attending a choir rehearsal. Try to imagine the sound of an all children's choir singing the first verse of a song.

- ○ 1 - No Image Present at All (1)
- ○ 2 (2)
- ○ 3 (3)
- ○ 4 (4)
- ○ 5 (5)
- ○ 6 (6)
- ○ 7 - As Vivid as the Actual Sound (7)

---

Q8 For the next item, consider attending a live concert. Try to imagine the sound of the band playing.

- 1 - No Image Present at All (1)
- 2 (2)
- 3 (3)
- 4 (4)
- 5 (5)
- 6 (6)
- 7 - As Vivid as the Actual Sound (7)

---

Q9 For the next item, listening to a rainstorm. Try to imagine the sound of gentle rain.

- 1 - No Image Present at All (1)
- 2 (2)
- 3 (3)
- 4 (4)
- 5 (5)
- 6 (6)
- 7 - As Vivid as the Actual Sound (7)

---

Q10 For the next item, consider attending class. Try to imagine the slow-paced voice of your English teacher.

- ○ 1 - No Image Present at All (1)
- ○ 2 (2)
- ○ 3 (3)
- ○ 4 (4)
- ○ 5 (5)
- ○ 6 (6)
- ○ 7 - As Vivid as the Actual Sound (7)

---

Q11 For the next item, consider seeing a live musical performance. Try to imagine the voice of the singer in the middle of a verse.

- ○ 1 - No Image Present at All (1)
- ○ 2 (2)
- ○ 3 (3)
- ○ 4 (4)
- ○ 5 (5)
- ○ 6 (6)
- ○ 7 - As Vivid as the Actual Sound (7)

---

Q12 For the next item, consider viewing a dance performance. Try to imagine the sound of the shoes on stage.

- 1 - No Image Present at All (1)
- 2 (2)
- 3 (3)
- 4 (4)
- 5 (5)
- 6 (6)
- 7 - As Vivid as the Actual Sound (7)

---

Q13 For the next item, consider a kindergarten class. Try to imagine the voice of the teacher reading a story to the children.

- 1 - No Image Present at All (1)
- 2 (2)
- 3 (3)
- 4 (4)
- 5 (5)
- 6 (6)
- 7 - As Vivid as the Actual Sound (7)

---

Q14 For the last item, consider driving a car. Try to imagine the sound of an upbeat pop song on the radio.

- 1 - No Image Present at All (1)
- 2 (2)
- 3 (3)
- 4 (4)
- 5 (5)
- 6 (6)
- 7 - As Vivid as the Actual Sound (7)

**End of Block: The Bucknell Auditory Imagery Scale - Vividness (BAIS-V)**

Start of Block: The Bucknell Auditory Imagery Scale - Control (BAIS-C)

Q15
The following scale is designed to measure auditory imagery, or the way in which you “think about sounds in your head.” For the following pairs of items you are asked to do the following: Read the first item (marked “a”) and consider whether you think of an image of the described sound in your head. Then read the second item (marked “b”) and consider how easily you could change your image of the first sound to that of the second sound and hold this image.

Rate how easily you could make this change using the “Ease of Change Rating Scale.” If no images are generated, give a rating of 1. Please read “a” first and “b” second for each pair. It may be necessary to cover up “b” so that you focus first on “a” for each pair.

Please feel free to use all of the levels in the scale when selecting your ratings.

For the first pair, consider attending a choir rehearsal. Imagine the sound of an all children's choir singing the first verse of a song.

AND THEN

An all-adults choir singing the second verse of the song.

- ○ 1 - Image Did not Change (1)
- ○ 2 (2)
- ○ 3 (3)
- ○ 4 (4)
- ○ 5 (5)
- ○ 6 (6)
- ○ 7 - Extremely Easy to Change the Image (7)

---

Q16 For the next pair, consider being at a night club. Imagine the sound of the music.

AND THEN

The music is accompanied by cheers from the crowd.

- ○ 1 - Image Did not Change (1)
- ○ 2 (2)
- ○ 3 (3)
- ○ 4 (4)
- ○ 5 (5)
- ○ 6 (6)
- ○ 7 - Extremely Easy to Change the Image (7)

Q17 For the next pair, consider listening to a rainstorm. Imagine the sound of gentle rain.

AND THEN

The gentle rain turns into a violent thunderstorm.

- 1 - Image Did not Change (1)
- 2 (2)
- 3 (3)
- 4 (4)
- 5 (5)
- 6 (6)
- 7 - Extremely Easy to Change the Image (7)

Q18 For the next pair, consider driving in a car. Imagine the sound of an upbeat pop song on the radio.

AND THEN

The car grinds to a screeching halt.

- ○ 1 - Image Did not Change (1)
- ○ 2 (2)
- ○ 3 (3)
- ○ 4 (4)
- ○ 5 (5)
- ○ 6 (6)
- ○ 7 - Extremely Easy to Change the Image (7)

---

Q19 For the next pair, consider ordering something over the phone. Imagine the voice of an elderly clerk assisting you.

AND THEN

The elderly clerk leaves and the voice of a younger clerk is on the line.

- ○ 1 - Image Did not Change (1)
- ○ 2 (2)
- ○ 3 (3)
- ○ 4 (4)
- ○ 5 (5)
- ○ 6 (6)
- ○ 7 - Extremely Easy to Change the Image (7)

Q20 For the next question, consider seeing a live musical performance. Imagine the voice of the singer in the middle of the verse.

AND THEN

The singer now reaches the end and holds the final note.

- 1 - Image Did not Change (1)
- 2 (2)
- 3 (3)
- 4 (4)
- 5 (5)
- 6 (6)
- 7 - Extremely Easy to Change the Image (7)

Q21 For the next appointment. Imagine the loud sound of the dentist's drill.

AND THEN

The drill stops and you can now hear the noise of the drill being placed on the bench.

- ○ 1 - Image Did not Change (1)
- ○ 2 (2)
- ○ 3 (3)
- ○ 4 (4)
- ○ 5 (5)
- ○ 6 (6)
- ○ 7 - Extremely Easy to Change the Image (7)

---

Q22 For the next pair, consider the beginning of the song "Happy Birthday." Imagine the sound of a trumpet beginning the piece.

AND THEN

The trumpet stops and a violin continues the piece.

- 1 - Image Did not Change (1)
- 2 (2)
- 3 (3)
- 4 (4)
- 5 (5)
- 6 (6)
- 7 - Extremely Easy to Change the Image (7)

Q23 For the next pair, consider attending a live concert. Imagine the sound of the band playing.

AND THEN

The band stops but the sound of the drums solo continues.

- ○ 1 - Image Did not Change (1)
- ○ 2 (2)
- ○ 3 (3)
- ○ 4 (4)
- ○ 5 (5)
- ○ 6 (6)
- ○ 7 - Extremely Easy to Change the Image (7)

---

Q24 For the next pair, consider attending a dance performance. Imagine the sound of the shoes against the stage.

AND THEN

The sound of the shoes speeds up and gets louder.

- 1 - Image Did not Change (1)
- 2 (2)
- 3 (3)
- 4 (4)
- 5 (5)
- 6 (6)
- 7 - Extremely Easy to Change the Image (7)

Q25 For the next pair, consider being at a live rugby game. Imagine the cheer of the crowd as a player scores.

AND THEN

The crows boos as the referee disallows the try.

- ○ 1 - Image Did not Change (1)
- ○ 2 (2)
- ○ 3 (3)
- ○ 4 (4)
- ○ 5 (5)
- ○ 6 (6)
- ○ 7 - Extremely Easy to Change the Image (7)

---

Q26 For the next item, consider a kindergarten class. Imagine the voice of the teacher reading a story to the children.

AND THEN

The teacher stops reading to talk to another teacher.

- 1 - Image Did not Change (1)
- 2 (2)
- 3 (3)
- 4 (4)
- 5 (5)
- 6 (6)
- 7 - Extremely Easy to Change the Image (7)

Q27 For the next pair, consider attending class. Imagine the slow-paced voice of your English teacher.

AND THEN

The pace of the teacher's voice getting faster at the end of class.

- 1 - Image Did not Change (1)
- 2 (2)
- 3 (3)
- 4 (4)
- 5 (5)
- 6 (6)
- 7 - Extremely Easy to Change the Image (7)

Q28 For the last pair, consider being at the beach. Imagine the sound of waves crashing against nearby rocks.

AND THEN

The waves are drowned out by the loud sound of a boat's horn at sea.

- ○ 1 - Image Did not Change (1)
- ○ 2 (2)
- ○ 3 (3)
- ○ 4 (4)
- ○ 5 (5)
- ○ 6 (6)
- ○ 7 - Extremely Easy to Change the Image (7)

**End of Block: The Bucknell Auditory Imagery Scale - Control (BAIS-C)**

---

Start of Block: Vividness of Visual Imagery Questionnaire - (VVIQ)

Q29 Try to imagine some relative or friend whom you frequently see (but who is not with you presently.) Consider the image that comes before your mind's eye with relation to: The precise body language and gait when walking.

- ○ 1 - No Image Present at All (1)
- ○ 2 (2)
- ○ 3 (3)
- ○ 4 (4)
- ○ 5 (5)
- ○ 6 (6)
- ○ 7 - As Vivid as Normal Vision (7)

Q30

For each of the items described below, please provide a rating that describes the vividness of the image in your 'mind's eye'.

Try to imagine some relative or friend whom you frequently see (but who is not with you presently.) Consider the image that comes before your mind's eye with relation to: The exact contour of the face, head, shoulders and body.

- ○ 1 - No Image Present at All (1)
- ○ 2 (2)
- ○ 3 (3)
- ○ 4 (4)
- ○ 5 (5)
- ○ 6 (6)
- ○ 7 - As Vivid as Normal Vision (7)

Q31 Try to imagine some relative or friend whom you frequently see (but who is not with you presently.) Consider the image that comes before your mind's eye with relation to: The characteristic poses of the head, attitudes of the body etc.

- 1 - No Image Present at All (1)
- 2 (2)
- 3 (3)
- 4 (4)
- 5 (5)
- 6 (6)
- 7 - As Vivid as Normal Vision (7)

---

Q32 Try to imagine some relative or friend whom you frequently see (but who is not with you presently.) Consider the image that comes before your mind's eye with relation to: The different colours worn in some familiar clothes.

- 1 - No Image Present at All (1)
- 2 (2)
- 3 (3)
- 4 (4)
- 5 (5)
- 6 (6)
- 7 - As Vivid as Normal Vision (7)

---

Q33 Try to visualise the rising sun. Consider carefully the picture that comes before your mind's eye when: The sun is rising above the horizon into a hazy sky.

- 1 - No Image Present at All (1)
- 2 (2)
- 3 (3)
- 4 (4)
- 5 (5)
- 6 (6)
- 7 - As Vivid as Normal Vision (7)

---

Q34 Try to visualise the rising sun. Consider carefully the picture that comes before your minds eye when: The sky clears and surrounds the sun with blueness.

- 1 - No Image Present at All (1)
- 2 (2)
- 3 (3)
- 4 (4)
- 5 (5)
- 6 (6)
- 7 - As Vivid as Normal Vision (7)

---

Q35 Try to visualise the rising sun. Consider carefully the picture that comes before your minds eye when: A storm in which flashes of lightening appears.

- 1 - No Image Present at All (1)
- 2 (2)
- 3 (3)
- 4 (4)
- 5 (5)
- 6 (6)
- 7 - As Vivid as Normal Vision (7)

---

Q36 Try to visualise the rising sun. Consider carefully the picture that comes before your mind's eye when: A rainbow appears.

- 1 - No Image Present at All (1)
- 2 (2)
- 3 (3)
- 4 (4)
- 5 (5)
- 6 (6)
- 7 - As Vivid as Normal Vision (7)

---

Q37 Try to imagine the front of a shop you often visit. Consider the picture that comes before your mind's eye when: You observe the overall appearance of the shop from the opposite side of the road.

- 1 - No Image Present at All (1)
- 2 (2)
- 3 (3)
- 4 (4)
- 5 (5)
- 6 (6)
- 7 - As Vivid as Normal Vision (7)

---

Q38 Try to imagine the front of a shop you often visit. Consider the picture that comes before your mind's eye when: You observe a window display including the colours, shape and details of individual items for sale.

- 1 - No Image Present at All (1)
- 2 (2)
- 3 (3)
- 4 (4)
- 5 (5)
- 6 (6)
- 7 - As Vivid as Normal Vision (7)

---

Q39 Try to imagine the front of a shop you often visit. Consider the picture that comes before your mind's eye when: You are near the entrance. The colour, shape and details of the door.

- 1 - No Image Present at All (1)
- 2 (2)
- 3 (3)
- 4 (4)
- 5 (5)
- 6 (6)
- 7 - As Vivid as Normal Vision (7)

---

Q40 Try to imagine the front of a shop you visit often. Consider the picture that comes before your mind's eye when: You enter the shop and go to the counter. The counter assistant serves you. Money changes hands.

- 1 - No Image Present at All (1)
- 2 (2)
- 3 (3)
- 4 (4)
- 5 (5)
- 6 (6)
- 7 - As Vivid as Normal Vision (7)

---

Q41 Try to imagine a country scene which involves trees, mountains and a lake. Consider the picture that comes before your mind's eye when: You observe the contours of the landscape.

- 1 - No Image Present at All (1)
- 2 (2)
- 3 (3)
- 4 (4)
- 5 (5)
- 6 (6)
- 7 - As Vivid as Normal Vision (7)

---

Q42 Try to imagine a country scene which involves trees, mountains and a lake. Consider the picture that comes before your mind's eye when: You observe the colour and shape of the trees.

- 1 - No Image Present at All (1)
- 2 (2)
- 3 (3)
- 4 (4)
- 5 (5)
- 6 (6)
- 7 - As Vivid as Normal Vision (7)

---

Q43 Try to imagine a country scene which involves trees, mountains and a lake. Consider the picture that comes before your mind's eye when: You observe the colour and shape of the lake.

- 1 - No Image Present at All (1)
- 2 (2)
- 3 (3)
- 4 (4)
- 5 (5)
- 6 (6)
- 7 - As Vivid as Normal Vision (7)

---

Q44 Try to imagine a country scene which involves trees, mountains and a lake. Consider the picture that comes before your mind's eye when: You observe a strong wind blowing against the trees and lake causing waves.

- 1 - No Image Present at All (1)
- 2 (2)
- 3 (3)
- 4 (4)
- 5 (5)
- 6 (6)
- 7 - As Vivid as Normal Vision (7)

**End of Block: Vividness of Visual Imagery Questionnaire - (VVIQ)**

---

Start of Block: Block 4

Q45 Would you like to receive, via email, a summary of research findings after they have been produced?

◯ Yes (1)

◯ No (2)

---

*Display This Question:*

*If Would you like to receive, via email, a summary of research findings after they have been produced? = Yes*

Q46 Please enter your email address so that we can send you a summary of the research findings. Please be assured that this information will be treated as confidential. Your email address will not be disclosed to any third parties.

________________________________________________________________

---

Q50 We are planning to carry out a further investigation, which will study possible links between mental imagery and pupillometry. Participating in this stage of the research will involve visiting the School of Psychology at the University of Auckland, in order to carry out tasks that assess the ability to perceive and remember colours. Performing these tasks will take approximately 15 minutes of your time. Would you like to receive further information via email, about this study, including information about how to volunteer to take part?

◯ Yes (1)

◯ No (2)

---

*Display This Question:*

*If We are planning to carry out a further investigation, which will study possible links between men... = Yes*

Q47
Please enter your email address here. Please be assured  that this information will be treated as confidential. Your email  address will not be disclosed to any third parties.lick to write the question text

________________________________________________________________

End of Block: Block 4
